# Protective Anti-HBs Antibodies and Response to a Booster Dose in Medical Students Vaccinated at Childhood

**DOI:** 10.3390/vaccines11081326

**Published:** 2023-08-05

**Authors:** Luca Coppeta, Cristiana Ferrari, Greta Verno, Giuseppina Somma, Marco Trabucco Aurilio, Luca Di Giampaolo, Michele Treglia, Andrea Magrini, Antonio Pietroiusti, Stefano Rizza

**Affiliations:** 1 Department of Biomedicine and Prevention, University of Rome Tor Vergata, 00133 Rome, Italy; luca.coppeta@ptvonline.it (L.C.); greta.verno@ptvonline.it (G.V.); giuseppina.somma@ptvonline.it (G.S.); michele.treglia@uniroma2.it (M.T.); andrea.magrini@uniroma2.it (A.M.); pietroiu@uniroma2.it (A.P.); 2 Faculty of Medicine, University “Nostra Signora del Buon Consiglio”, Tirana 1000, Albania; rizza@med.uniroma2.it; 3 Department of Medicine and Health Sciences “V. Tiberio”, University of Molise, 86100 Campobasso, Italy; marco.trabuccoaurilio@unimol.it; 4 Department of Occupational Medicine, University of Chieti “G. D’Annunzio”, 66100 Chieti, Italy; luca.digiampaolo@unich.it; 5 Department of System Medicine, University of Rome Tor Vergata, 00133 Rome, Italy

**Keywords:** HBV, vaccination, booster dose, medical students, circadian rhythm, immunological memory

## Abstract

The immune system in humans is regulated by the circadian rhythm. Published studies have reported that the time of vaccination is associated with the immune response to vaccine for some pathogens. Our study aimed to evaluate the association between time of dose administration of challenge HBV vaccine and seroconversion for anti-HBs in medical students vaccinated at birth who were found to be unprotected at pre-training screening. Humoral protection for HBV was assessed in 885 medical students vaccinated during childhood. In total, 359 (41.0%) of them showed anti-HBs titer < 10 UI/mL and received a challenge dose of HBV vaccine followed by post-vaccination screening 30–60 days later. The challenge dose elicited a protective immune response (anti-HBs IgG titer > 10 UI/mL) in 295 (83.8%) individuals. Seroconversion was significantly associated with female gender and time of vaccination after controlling for age group and nationality at logistic regression analysis. Students who received the booster dose in the morning had a higher response rate than those who received the vaccine in the afternoon (OR 1.93; 95% C.I. 1.047–3.56: *p* < 0.05). This finding suggests that morning administration of the HBV booster may result in a better immune response in susceptible individuals.

## 1. Introduction

The succession of day and night rhythms is generated by an internal biological clock, called the circadian clock, which is synchronized to the 24 h day by environmental cues, primarily the light–dark cycle. The light–dark cycle therefore regulates many biological functions in humans, such as the sleep–wake cycle, body temperature, blood pressure and metabolism. In addition, regular sleep has a beneficial effect on the functioning of the immune system, and the production of pro-inflammatory cytokines in response to infection affects sleep regulation [1]. Therefore, chronic sleep deprivation may increase the risk of some chronic diseases associated with low-grade inflammation, such as diabetes, atherosclerosis and neurodegeneration [2].

There is compelling evidence that the immune response of innate and adaptive immunity to pathogens in terms of susceptibility, clinical presentation and severity is closely related to circadian rhythm, either quantitatively or qualitatively [3].

The number of circulating hematopoietic cells and leukocytes in humans increases during the night, and the homing of circulating lymphocytes follows a rhythmic process [4]. Furthermore, antibody production in response to antigenic stimuli varies with the rhythm of melatonin secretion in mice, demonstrating the influence of hormones and neuro-mediators that reflect the activity of the central pacemaker [5]. Interestingly, a diurnal variation in the rate of positive test results and viral load for SARS-CoV-2 has been demonstrated [6,7].

Sleep quality may also influence IgG antibody titer and the duration of immunity to various vaccines, according to early studies suggesting that sleep may allow the immune system to respond more effectively to the vaccine stimulus [8,9].

It has been shown that the response to the influenza vaccine is significantly affected by the time of administration [10], and that morning vaccination results in greater immune responses to hepatitis A and influenza vaccines [11].

Circadian variation may have a significant impact on the response to vaccination in people who work or train in areas where there is a higher risk of HBV infection, such as healthcare workers and medical students [12,13,14,15,16,17]. In Italy, HBV vaccination is mandatory at birth age for all individuals born after the year 1991, but published studies have shown that the percentage of young individuals who are unprotected at the time of their first employment screening is high [12,14], raising questions about the effectiveness and duration of protection in those vaccinated at age <12 months and highlighting the need of a booster dose in these unprotected individuals. The response to a booster dose is generally high in previously vaccinated individuals, demonstrating the persistence of immune memory; however, a lack of response may occur in a variable percentage of individuals, posing a serious safety issue for these individuals. In this regard, a recent paper [18] reported that in South Africa, more than half of young healthcare students were not immune to HBV before receiving the recommended “booster” vaccine. However, a significant proportion (7%) remained unprotected after vaccination, highlighting that without confirmation of immune status, young people cannot be assumed to be protected after a “booster”. Therefore, the optimization of the procedures aimed to improve the response the booster dose is crucial, in order to minimize the rate of unprotected people. In this light, we conducted a retrospective hypothesis-generating study to evaluate the influence of administration timing on booster dose response in a group of medical students vaccinated at birth who underwent pre-employment screening at the Policlinico of Tor Vergata.

## 2. Materials and Methods

This retrospective hospital-based study, conducted by the Tor Vergata Occupational Medicine Service in 2023, was approved by the Independent Ethics Committee of the University Hospital PTV (Policlinico of Tor Vergata) in Rome, Italy. Records were extracted from all medical students born after 1 January 1991 who attended their pre-training screening in 2022. Data from individuals with diabetes, chronic liver disease, renal insufficiency, heart failure, a positive history of any form of cancer and positive blood tests for HIV were excluded from the analysis. We also excluded students who had a positive HBsAg result because we considered them to be affected by chronic HBV hepatitis, as well as subjects who tested positive to anti-HBc-IgG because we considered them to have had a previous infection. For each subject, childhood vaccination was verified by collecting a copy of the vaccination booklet, including foreign students. Those who could not provide a written vaccine record, were excluded from the study and were fully vaccinated (three-dose series on a 0-, 1- and 6-month schedule). To evaluate the immune status, each subject was venipunctured in one of the veins of the arm (the cephalic vein or median cubital vein or basilic vein) and a blood sample of 10 mL was collected. The blood tubes were sent to the laboratory for analysis to determine the level of anti HBsAg antibodies. Serological evaluation was performed using the ECLIA (Electro Chemi Luminescence Immuno-Assay) method.

Students were divided into two groups according to their anti-HBs test result: subjects with anti-HBs IgG levels ≥10 mIU/mL were considered protected, whereas subjects with anti-HBs IgG levels <10 mIU/mL were considered unprotected.

Since the Hospital has a mandatory policy for HBV vaccination, all subjects without serological evidence of immunity had received a booster dose and a subsequent blood test for anti-HBs 1–2 months after the vaccination to assess the immune response, according to the recommendations of the Italian Ministry of Health. The following covariates were collected for the study protocol: age, sex, anti-HBs IgG, anti-HBc IgG level and HBsAg status. 

The vaccination service in the occupational medicine department works in two shifts: morning (9.00–11.00 a.m.) and afternoon (2.00–4.00 p.m.), so the study subjects, accordingly with their preference or availability, randomly received the booster dose during one of these two shifts.

The post-vaccination test of the two sample groups (morning or afternoon, depending on the time of day when the booster dose was administered) was performed in the same time slot (7.30 to 10.30 a.m.) for all study participants.

### Statistical Analyses

Subject characteristics are reported as numbers and percentages, depending on the type of variable. Differences in percentages between groups were assessed using the Χ_2_ test or Fisher’s exact test, as appropriate. Each continuous variable was tested for normality using the Shapiro—Wilk goodness of fit test. Pearson’s test was used to assess the correlation between continuous variables. Finally, simple and multiple logistic regression analysis was performed to explore the associations between HBs-IgG-titer after booster (protective vs. non-protective) and all factors significantly correlated with the dependent variable. A *p* value < 0.05 was considered statistically significant. All analyses were performed with SPSS version 25.0 for Windows.

## 3. Results

We analyzed the records of 885 medical students. After screening, we excluded seven individuals who tested positive for HBs Ag and two individuals because they tested positive for anti-HBc. All these individuals were born in Italy. We also excluded 1 person because of overt diabetes, so the study included 875 participants.

The mean age of the study participants was 21.7 ± 1.69 years (see Table 1). Most subjects were female and of Italian nationality. Table 2 shows the characteristics of the populations according to HBV titers at baseline. Overall, a protective anti-HBs level was found in 516 (59.0%) of the study subjects. Female students were more protected than their male counterparts (*p* < 0.05), but the *t*-test did not reveal a significant difference in mean titers between genders. Notably, baseline anti-HBs level was inversely related to the age at which the study was conducted (r = −0.321, *p* < 0.01).

**Figure 1:**
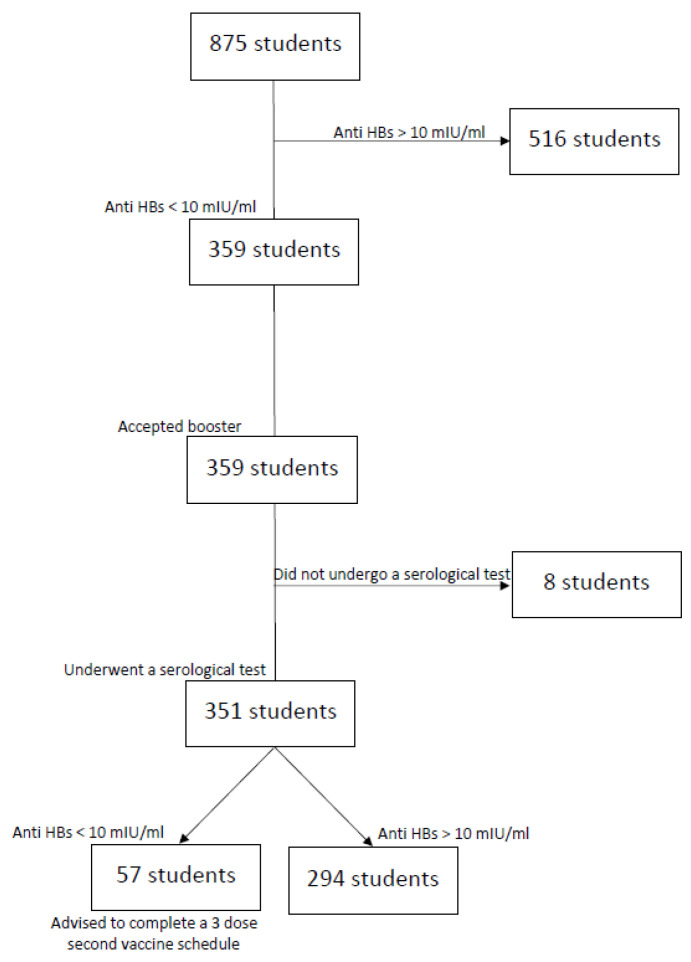
Flowchart describing the study protocol.

Unprotected subjects (n = 359) received a booster dose followed by a blood test 30–60 days later. A total of 351 students returned for post-booster evaluation, while 8 subjects did not complete the screening and were excluded from the analysis. Of the students who received the booster dose, 294 individuals (83.8%) developed an anti-HBs titer >10 UI/mL and were therefore considered to have immune memory and were classified as “responders”. In contrast, those who did not show a protective antibody level were classified as “unprotected” and were advised to complete a second three-dose vaccination schedule, in accordance with current recommendations (see Figure 1). The mean interval between booster administration and blood sampling was 34.0 ± 5.5 days (range of 31–59 days). Post-booster delay was not associated with antibody titers by Pearson’s test (r = 0.18; *p* = 0.12). There was a significant gender difference in seroconversion (79.1% vs. 86.5% in male and female students, respectively; *p* < 0.05). This apparent difference between female and male subjects could potentially be confounded by different lifestyle factors (diet, alcohol consumption, vigorous exercise), which are known to influence immune status. Interestingly, the administration of a booster dose in the morning was associated with both a higher rate of protection and a higher anti-HBs titer compared to receiving the additional dose in the afternoon.

Finally, using a logistic regression model, we found that morning administration had a 1.93-fold significant odd ratio (OR = 1.93, 95% CI = 1.047–3.561, *p* < 0.05) for the development of a protective HBV titer after booster dose compared to afternoon administration, after adjusting for sex (see Table 3).

## 4. Discussion

Hepatitis B is a viral disease of global importance. Following the introduction of a routine neonatal HBV vaccination program, the incidence of chronic HBV infection has fallen dramatically to <2% [19]. However, the duration of protection is still unknown, and the question of the need for a booster dose in vaccinated individuals remains a challenge. The results of long-term follow-up studies showing no significant breakthrough infections in the vaccinees over a 30-year period, and the response to a challenge dose accounting a durable immunological memory in most vaccinated individuals, are reassuring for durable protection in the general population [20,21]. Although some reports have described the reduced incidence of hepatitis B and high immunogenicity in the young Italian population [22], these findings should be interpreted with caution when assessing protection in subjects at moderate to high risk of HBV infection, such as medical students, for whom even a small percentage of non-responders represents a serious threat. Studies such as the present one, conducted in young individuals before they enter training, provide a unique opportunity to assess the persistence of humoral and cellular immunity and to explore the most effective strategies for achieving complete immunization in medical students who will soon be exposed to high-risk activities.

To our knowledge, this is the first study to show that the time of day of vaccination affects the antibody responses to HBV vaccine boosters. In fact, we found an almost 2-fold higher humoral response to a challenge dose in those operators vaccinated in the morning compared to those vaccinated in the afternoon, regardless of age and sex. The results of our study, although derived from a relatively small sample, suggest a role for the circadian clock in modulating the immune response to HBV vaccination.

Our main finding is consistent with previous evidence that the regulation of adaptive immune responses is controlled by circadian rhythms. Previous reports indicated that hepatitis A and B vaccination is immune-enhanced by efficient sleep [9,23,24] and described a relationship between vaccination timing and antibody production in influenza [10]. In addition, the time of day has recently been shown to influence anti-tuberculosis vaccination with bacillus Calmette–Guérin (BCG), suggesting that morning vaccination may confer a much greater immunological advantage than evening vaccination [25]. Therefore, the optimal time for vaccination appears to be regularly around or just before the behavioral activity phase, and in humans this may be the early morning.

The immunopathological mechanisms underlying the different immunological responses to the booster dose were not investigated in the present study and therefore any interpretation of the study results remains speculative. However, the different influence of the time of day of vaccination on the serological response after HBV vaccination could be an effect of circadian changes through immunological cells regulations. Indeed, it is well known that circadian rhythms, a potent regulator of immune function, can alter the magnitude of immune responses [26,27,28,29]. Daily variations in antibody production after vaccination have been reported in both humans [25] and in mice [30], but studies investigating the pathophysiological processes and the relative contributions of circadian rhythms in different aspects of the immunological response are very few. However, we can speculate that rhythmic expression of clock genes (Per1, Per2, Rev-ERBα, BMAL1 and Clock), which are often modulated by circadian oscillation of the endogenous clock, leisure activities, or diet, may influence the adaptive immunity and vaccination responses. Accordingly, circadian changes in the expression of clock genes have been demonstrated in the antigen presenting cells (APCs) of different tissues (macrophages, dendritic cells and B cells) in response to a light–dark cycle [31]. The role of APCs in the vaccine-induced response and in the recruitment of memory cells may be a key element in explaining the different pattern of response to HBV vaccine found in our study. Again, according to published findings, all the T cells subpopulations (including central and effector memory) show a 24 h pattern in their blood cell counts, opposite to the cortisol rhythm, with a peak at night and a trough in the afternoon [11].

Notably, it is also possible that the memory cell response, influenced by the endogenous rhythm of cortisol and inflammatory cytokines, is less able to respond to antigenic stimuli at a certain hour of the day (afternoon), when the number and the functionality of B and T lymphocytes are at their lowest. Accordingly, a very recent important article [32], using a combination of experimental and mathematical modeling approaches, provides mechanistic insights into a diurnal regulation of vaccination responses. Specifically, the authors reported that in mice, the circadian clock is able to control and support an adaptive immune response at a physiological level over an extended period of time, preventing it from either overreacting or diminishing. An enhanced immune response just after the halfway point of the rest period and before the onset of the behavioral activity period may make stimulated individuals more likely to encounter pathogens, for example during foraging or social interactions.

Furthermore, it is possible that the suppression of melatonin release in response to light exposure, may affect the functions of T and B lymphocytes and the production of antibodies in response to antigens, explaining the increased antibody production after morning vaccination found in our study [5,33]. Again, according to recent findings, all the T cells subpopulations (including central and effector memory) show a 24 h pattern in their blood cell counts in opposite to cortisol rhythm, with a peak at night and a trough in the afternoon [34,35].

Interestingly, similar results have also been reported in population older than those recruited in this study. In particular, higher anti-influenza antibody titers have been reported in adults vaccinated in the morning compared to those vaccinated in the afternoon [10,11]. Conversely, in a study of a large sample of healthcare workers, the magnitude of the anti-spike response was higher after afternoon versus morning administration of the COVID-19 vaccine [36]. According to the authors, these conflicting results may reflect differences in the immune status with respect to the above-mentioned studies (primary response for COVID-19 versus memory responses to influenza vaccination). Furthermore, in a recent study investigating morning versus afternoon vaccine response in a sample of healthy young adults, the timing of vaccination was not associated with immune antibody response. As suggested by the authors, the effects of time of day may be limited to thymus-dependent antigenic vaccines, which involve more components than the thymus-independent vaccine response, each of which may be influenced by circadian rhythm effects [37]. Furthermore, this observation was made in a study of only 136 individuals (75 healthy young adults and 61 parents), of whom the mean age of the 61 parents was 41.4 years old. Overall, these characteristics make this report quite different from our main finding.

Unfortunately, due to the retrospective nature of the study, we do not have data on the functional indices of the immune system of these study participants. However, it is known that in immunocompetent individuals, immunity to HBV outlasts the presence of vaccine-induced antibodies, resulting in long-term protection against the disease, even in those without detectable anti-HBs titers [38,39].

With regard to another factor evaluated in the present study, we found that approximately half of the medical students vaccinated at birth lacked protective antibody titers 10–24 years after the three-dose vaccination series. In addition, we observed that antibody titers were inversely related to age, in line with previous findings that antibody titers often decline over time [40]. We also reported a slightly, but not significantly, higher level of protection in females compared to males. It is known that the competence of the immune response is sex dysmorphic, contributing to sex differences in the pathogenesis of infectious diseases. This gender effect occurs because estrogens typically promote an immunostimulatory effect, whereas androgens are immunosuppressive [41,42,43,44]. Women often have greater humoral and cell-mediated immune responses than men after antigenic stimulation, including vaccination and infection [45]. Similarly, women have a more pronounced immune response than men after seasonal influenza vaccination [46]. This finding may be due to the greater absolute number of CD4-positive (CD41) lymphocytes in women than in men [47]. Similarly, women have a greater production of TH1 cytokines [48,49,50], resulting in a greater release of neutralizing antibodies after vaccination. Moreover, the apparent difference between female and male subjects could potentially be confounded with different lifestyle factors (diet, alcohol consumption, intensive sport) that are known to affect the adaptive immunity and vaccination responses.

Notably, the majority of students who tested unprotected at baseline developed a protective anti-HBs title following administration of a challenge dose, confirming the presence of durable protection in those vaccinated at birth [51,52].

In addition, recent long-term follow-up studies have highlighted the occurrence of breakthrough HBV infection, as evidenced by seroconversion to anti-HBc in the absence of clinically significant infection or viral carriage [51,53].

Although the prevention of acute clinical HBV infection and viral carriage is the main public health goal of HBV vaccination, this may not be sufficient for medical students. In these individuals, the presence of even a small proportion of unprotected individuals poses a serious risk of further professional infection. The lack of an anamnestic response needs to be better understood and long-term follow-up studies are needed to show how long immune memory lasts and whether, when and at what age booster doses may be needed to ensure continued protection.

Our study has some obvious limitations. First, we do not have data on the serological response to the first series of childhood vaccines, which has been associated with the development of immunological memory in published studies [44]. Second, due to retrospective nature of the study, the study protocol did not assess duration and quality of sleep over the night before vaccination; this limitation is important because previous studies have highlighted the relationship between sleep and immunological response to vaccines [1]. Third, the gender composition of the study group was unbalanced, since the vast majority of the vaccinated group was female. As a result, the generalization of our findings to male operators is limited. Fourth, the study population is relatively small, it includes only a specific type of participants (medical students) and only few independent variables; therefore, our results cannot be generalized. Moreover, no sample size or randomization was performed as all students who underwent pre-training screening during the study period were included. Finally, blinding would have made the study more robust.

## 5. Conclusions

Our study provides additional data on the role of circadian rhythms in HBV vaccine response. Based on the results of our study, the vaccine administration in the morning times rather than in the afternoon should be recommended in vaccinations schedules. Although the main result of this work is a simple association, although controlled for important confounding factors, we can speculate that circadian rhythm may have modulated the immune response to HBV by affecting the function of immune cells. It is possible that the response of memory cells, influenced by the endogenous rhythm of cortisol and inflammatory cytokines, is less able to respond to antigenic stimuli at a certain hour of the day (afternoon), when the number and functionality of B- and T-lymphocytes are at their lowest. Therefore, by revealing a kind of rhythmicity in vaccination responses with a booster dose, our findings are of direct relevance to public health. In particular, in cases of limited vaccine availability or a compelling need for high levels of protective immunity, as observed in the SARS-CoV-2 pandemic, successful enhancement of rhythmicity could improve vaccination regimes. However, many questions remain to be solved regarding the mechanisms underlying the bidirectional link between the circadian clock and the immune system. Longitudinal studies are warranted to better understand the immune response following vaccination in relation to chronobiological variables.

## Figures and Tables

**Table 1:** Baseline general characteristics of study population.

	N	Percent (%)
Total number	875	
Mean age (SD)	21.7 ± 1.69	
Anti-HBs titer		
>10 UI/mL	516	59.0
<10 UI/mL	359	41.0
Titer after booster		
>10 UI/mL	294	83.8
<10 UI/mL	57	16.2

**Table 2:** Main characteristics of the study population who tested protective (anti HBs > 10 mIU/mL) after booster.

	N	Percent (%)	
Sex			
Male	102	86.5	*p* < 0.05
Female	192	79.1	
Time since first dose			
>22 years	182	84.3	*p* = n.s.
<22 years	112	83.0	
Nationality			
Italian	260	83.1	*p* = n.s.
Foreign	34	89.5	
Time of administration			
Morning	217	86.1	*p* < 0.05
Afternoon	77	77.8	

**Table 3:** Multivariate logistic regression model with anti-HBs titer > 10 mIU/mL as dependent variable.

Variables	Odd Ratio	95% CI for OR	*p* Value
Sex (male)	0.563	0.314–1.007	n.s.
Time since first dose (>22 years)	1.013	0.558–1.839	n.s.
Nationality (Italian)	0.514	0.172–1.535	n.s.
Time of administration (morning)	1.931	1.047–3.561	<0.05

## Data Availability

Datasets used and/or analyzed during the current study are available from the corresponding author on reasonable request.
